# Workforce Safety Vulnerabilities in Pakistan’s Construction Sector

**DOI:** 10.5334/aogh.5077

**Published:** 2026-04-15

**Authors:** Muhammad Umair, Muhammad Uzair Yousuf

**Affiliations:** 1 Department of Economics, University of Karachi, Karachi 75270, Pakistan; 2 Department of Mechanical Engineering, NED University of Engineering and Technology, Karachi 75270, Pakistan

**Keywords:** construction industry, index value trend, Labour Force Survey, occupational injuries

## Abstract

*Background:* The construction workforce drives infrastructure growth in Pakistan but faces severe occupational safety and health (OSH) risks, including high injury rates and poor protection. These vulnerabilities threaten worker well-being and jeopardize sustainable development.

*Objective:* This study investigates OSH vulnerabilities among construction workers in Pakistan and identifies sociodemographic factors contributing to occupational injuries.

*Methods:* The Labour Force Survey (LFS), a nationally representative survey conducted by the Pakistan Bureau of Statistics, collects comprehensive data on employment, occupational injuries, and sociodemographic characteristics using standardized questionnaires. LFS data from 2001–02 to 2017–18 were analyzed using the index value trend method, documenting construction worker injuries from 151 cases (7.3% injury rate) in 2001–02 to 516 cases (8.6% injury rate) in 2017–18 across four provinces. Injuries were examined by age, province, education, occupation, unsafe acts, unsafe conditions, and treatment received.

*Findings:* Younger workers, those in rural areas, and low-skilled, less-educated blue-collar employees faced the highest risk of occupational injuries. Unsafe practices, including neglecting the use of protective equipment and improper handling of materials, combined with hazardous site conditions, were the primary contributors to accidents. Trends across provinces and occupational categories reveal persistent vulnerabilities, indicating that despite some minor improvements over time, significant risks in workplace safety remain.

*Conclusions:* Targeted OSH interventions, including safety training, mandatory compliance in construction projects, and formalization of informal labor, are essential. Integrating OSH measures with social protection programs can reduce injuries, improve workforce health, and support safer practices in Pakistan’s infrastructure development.

## Introduction

### Background of the study

The construction sector is crucial for infrastructure development in developing countries, providing labor for power plants, renewable installations, and transmission networks. Yet, it remains highly hazardous, with workers facing frequent injuries, limited social protection, and occupational illnesses. In Pakistan, where construction expansion supports economic growth and sustainability, these occupational safety and health (OSH) risks not only threaten worker well-being but also pose broader risks to project timelines, costs, and long-term infrastructure sustainability [1–3]. Unfortunately, construction companies have limited awareness and implementation of OSH practices, resulting in inadequate recognition and management of workplace hazards [4]. As a consequence, construction workers are frequently exposed to unsafe conditions, insufficient protective equipment, and weak safety supervision [5]. These challenges not only increase the risk of physical injuries but also contribute to psychosocial hazards such as safety concerns, exposure to stressful or traumatic events, and job insecurity. Such conditions can adversely affect workers’ health, while low safety awareness, limited training, and economic pressures often encourage risky behaviors. Ultimately, these combined risks lead to broader social consequences, including financial stress and job instability [6–10]. Therefore, promoting robust OSH practices is essential to protect lives and mitigate health risks among construction workers [11]. In addition, implementing preventive measures, services, and policies is critical for ensuring a safe and secure working environment [12].

**Figure 1:**
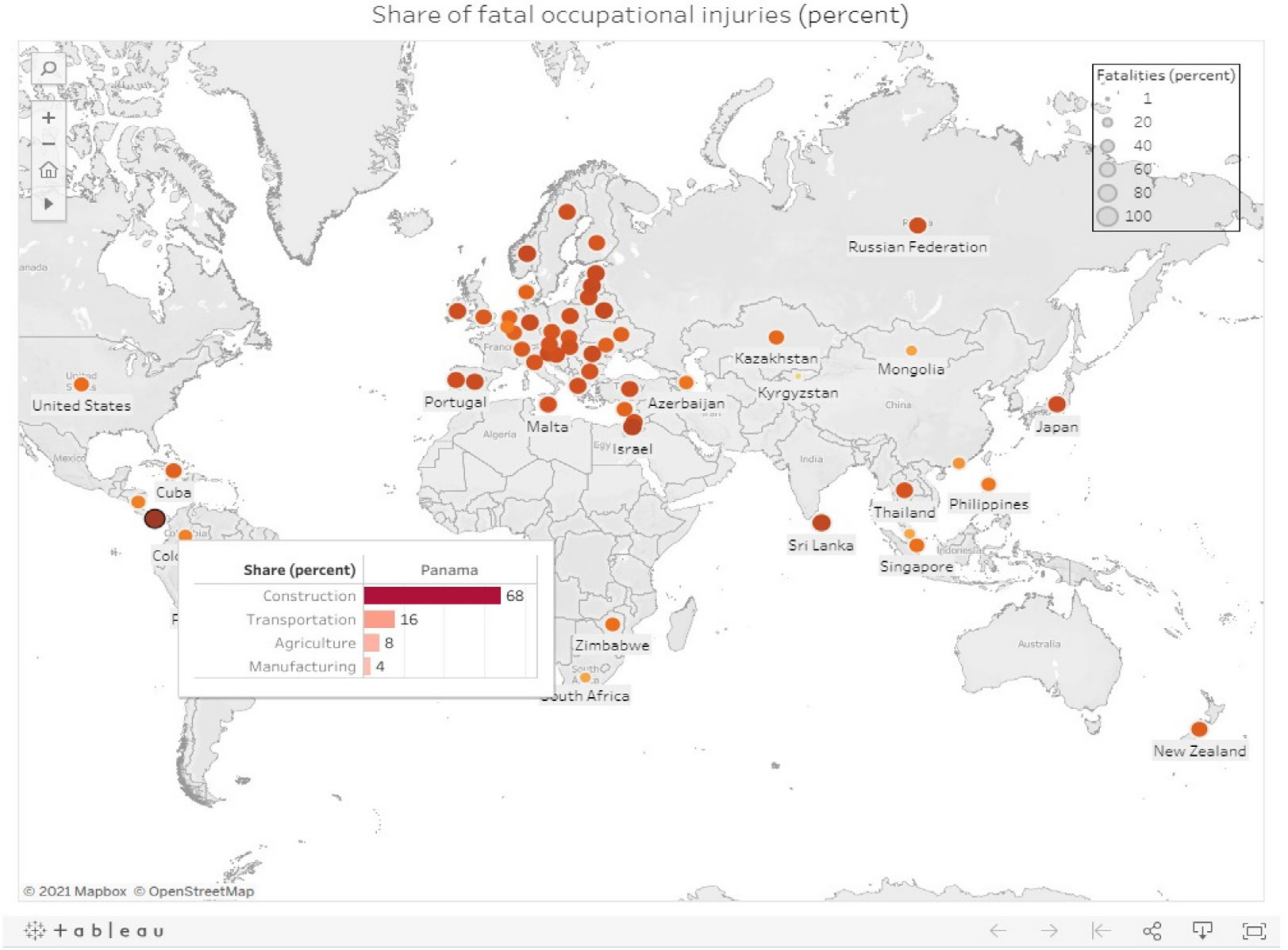
Fatal occupational injuries in the selected countries and sectors. Source: Tableau Public [11].

International Labour Organization (ILO) data [11], presented in Figure 1, compare sector-specific fatalities across construction, agriculture, manufacturing, and transportation in various countries. A more detailed analysis is available on the open-access Tableau Public^1^ platform developed by the authors. The figure highlights that Panama and Malta report the highest construction fatalities, whereas the Netherlands records the highest nonfatal incidents, emphasizing construction as the most hazardous sector in both developed and developing economies. Despite its significance, sector-wise fatality data remain unavailable for Pakistan, even though it hosts one of the largest labor forces in the world. ILO data further indicate that in Pakistan, occupational injuries reached 1,180 per 100,000 workers in 2002, with a fatal injury rate of 44 and a nonfatal injury rate of 1,136. However, this evidence is outdated; to the best of the authors’ knowledge, more recent data are unavailable due to the lack of updated reporting in the Labour Force Survey (LFS), which has not included detailed fatal injury information since 2006–07, despite earlier surveys providing more comprehensive injury-related data [13].

**Figure 2:**
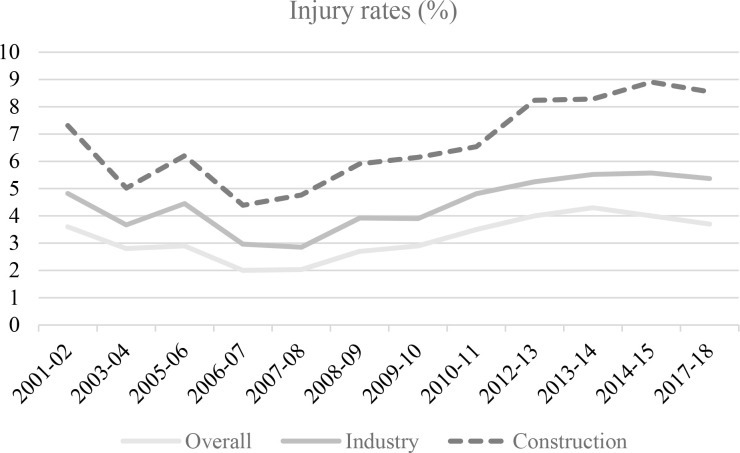
Trends in construction, industrial, and total occupational injury rates (%) in Pakistan. Source: [15].

In Pakistan, the construction sector accounts for 2.5% of the gross domestic product (GDP) and provides employment for 7.6% of the total workforce. Due to the inherently hazardous nature of construction activities, work-related injuries are particularly prevalent in this sector. Workers routinely face physical risks, including working at heights, handling heavy materials, and operating machinery, all of which substantially increase the likelihood of occupational injuries. This heightened vulnerability is evident in the OSH conditions of construction workers in Pakistan. Figure 2 presents a comparison of injury rates in the construction sector with those in the broader industrial sector and the national average over time. On average, the construction sector records an injury rate of approximately 6.7%, more than double the national average of 3.2%, underscoring the disproportionate safety risks faced by construction workers [14].

Unlike injuries caused by sudden accidents, occupational diseases develop gradually due to prolonged exposure to workplace hazards such as harmful substances, unsafe environments, and repetitive physical strain. Monitoring these diseases is often challenging because there is usually a significant time lag between exposure to occupational risk factors and the appearance of disease symptoms. According to Pakistan’s LFS 2017–18 [15], workers who experience either occupational injuries or work-related diseases are collectively classified as injured workers in the dataset. Notably, the trends in total, industrial, and construction injury rates in Pakistan have shown an upward trajectory since 2007–08. The ability of injured workers to return to work and resume normal activities largely depends on the severity of the injury. Severe injuries can result in extended absences from work and significant disruptions to daily activities, whereas minor injuries may require little or no medical treatment, allowing workers to return to work on the same day. Consequently, the duration of recovery and work absence varies according to the seriousness of the accident. In addition, the construction sector records a higher incidence of multiple injuries compared with other industrial sectors, reflecting the complex and hazardous nature of construction work environments.

### Framework

Construction workers face higher risks of severe injuries and fatalities than workers in many other industries, although comparisons are often limited by differences in reporting methods. The definition of a construction worker is also not always clear, particularly regarding whether it differs from workers employed in the construction industry [16]. Moreover, only a limited number of empirical studies have examined long-term trends in the socioeconomic and demographic characteristics of occupational injuries in the construction sector in Pakistan and globally.

Noman et al. [3] analyzed Pakistan’s LFS data from 2001–02 to 2017–18 using index value calculations. The study reported that the traditional farm (agriculture, forestry, hunting, and fishing) sector had the highest injury rates due to structural factors, rural workforce concentration, and linkages with modern sectors, while construction workers experienced the second-highest and steadily increasing injury rates, driven by risky employment, high unemployment, and employer negligence. Moreover, Umair and Naz [2] analyzed Pakistan’s LFS data from 2001–02 to 2017–18 to examine index value trends in the socioeconomic and demographic characteristics of injured internal migrant workers. Their findings show that males and individuals who previously resided in areas classified as rural but have since migrated to urban areas are most vulnerable to occupational injuries, likely due to greater exposure to risky construction tasks and limited access to training, safety equipment, and healthcare. Education and prior training reduce injury risks by improving awareness of safety practices. The study recommends stronger enforcement of labor laws and the development of targeted health and safety policies for vulnerable groups such as internal migrants and rural laborers. Similarly, Abbas [1] analyzed Pakistan’s LFS data from 2001–02 to 2012–13 using index values and Pearson correlation. The study found a rising trend of occupational injuries, especially in high-risk sectors like construction and agriculture, driven by limited medical and emergency facilities in rural areas. It underscores the need to improve rural health infrastructure and strengthen OSH measures, as highlighted by the ILO.

Apart from Pakistan, Rhee et al. [17] analyzed Korea’s Yearbooks of Industrial Accidents (2001–10) using index values and found a rising trend in construction-related fatalities, particularly among workers aged 45 and above. Unsar and Sut [18] examined Turkey’s SSI Statistical Yearbooks (2000–05) and reported higher occupational accident rates in construction, metal manufacturing, textile, and coal mining sectors, with elevated fatalities and permanent disabilities in construction. Their comparison with 15 European countries (1998–2001) emphasized that prevention strategies, employee cooperation, and a safety-focused culture can reduce occupational accidents. Shafique and Rafiq [19] analyzed Hong Kong data (2011–17) using ANOVA and found construction to be one of the riskiest sectors. Injuries were mainly from slips, trips, and falls, while fatalities were largely due to falls from heights. The study recommended safety management systems, audits, professional oversight, and advanced technologies like sensors, robotics, and automation to reduce occupational fatalities. Choi et al. [20] compared fatal construction injuries in the United States, South Korea, and China (2011–15). Falls from heights and being struck by objects were the most common causes. Comparative evidence shows that China records the highest average number of construction-related fatal occupational injuries (2,328), followed by the United States (881) and South Korea (533). However, South Korea has the highest mortality rate (17.9), exceeding the United States (9.4) and China (5.3), highlighting the importance of considering both absolute and relative risk measures. Fatalities rose annually in the United States (5.8%) but declined in China (7.1%) and South Korea (4.9%). Differences in workforce profiles and reporting systems, including potential underreporting, were noted.

In the context of construction literature, Shafique and Rafiq [19] employed ANOVA to review the recent trend of occupational accidents in Hong Kong over time. Some previous studies in the literature used index value analysis to assess the trend of occupational injuries, such as Noman et al. [3] and Abbas [1] for Pakistan, Rhee et al. [17] for Korea, and Unsar and Sut [18] for Turkey. Additionally, Umair and Naz [2] examined occupational injuries among internal migrant workers in Pakistan. Abbas [1] evaluated trends in occupational injuries using the index value method; however, the study primarily provided a general overview without offering a detailed examination of sociodemographic factors such as age, province, or education level of injured construction workers. Furthermore, critical aspects of accident causation, including unsafe acts by personnel and hazardous workplace conditions, were not investigated. Previous studies largely relied on statistical yearbooks and annual reports, which offered limited insights.

Notably, an in-depth and comprehensive discussion of construction injuries in Pakistan was lacking in the existing research. This study addresses this gap by providing a detailed and systematic analysis of occupational injury trends among construction workers in Pakistan using long-term national survey data. By applying index value analysis across multiple sociodemographic and occupational dimensions, the study offers a broader understanding of safety vulnerabilities within the construction sector. The novelty of this research lies in its comprehensive approach to examining construction injuries over an extended period, which allows for identifying patterns and structural risks that have not been sufficiently explored in previous studies. Such evidence is important for informing OSH policies and improving worker protection in Pakistan’s rapidly expanding construction and infrastructure development sector. The key contributions of this study include:

1. Provides a comprehensive assessment of the construction industry, worker vulnerability to OSH issues, and injury profiles.
2. Analyzes 12 waves of LFS data (2001–02 to 2017–18) to examine trends in construction worker injuries. The 12 waves of the LFS refer to the 12 rounds of nationally representative surveys conducted by the Pakistan Bureau of Statistics (PBS) from 2001–02 to 2017–18, which collect standardized data on employment, occupational injuries, and sociodemographic characteristics of the workforce.

## Materials and Methods

### Data extraction

This research utilized data from Pakistan’s LFS [15], which is collected, processed, compiled, and disseminated by the PBS to inform labor policies and their implementation. The LFS employs a stratified two-stage sampling design, which first divides the population into distinct strata, such as provinces or urban and rural areas, to ensure representation across key demographic and geographic groups. In the second stage, households within each stratum are randomly selected for participation. Data are collected through direct interviews with individuals in these households using questionnaires that capture comprehensive information on employment, occupational injuries, education, and other sociodemographic factors. This structured methodology ensures that the survey results are nationally representative and reliable for analyzing trends in OSH among construction workers. The study examined injury patterns in Pakistan’s construction industry using 12 waves of LFS data from 2001–02 to 2017–18. To focus on occupationally injured construction workers, the analysis refined the initial LFS samples for each wave to isolate relevant cases in the four provinces. The number of reported occupational injuries in the construction sector increased from 151 cases, corresponding to a 7.3% injury rate in 2001–02, to 515 cases, with an 8.6% injury rate in 2017–18, reflecting both the growing workforce and persistent safety challenges. The process begins with the initial LFS sample and systematically narrows it down: first by selecting data from the four provinces, then by including individuals aged 10 years and above, followed by those who are employed, and then specifically construction workers. Finally, the subset of injured construction workers is identified. This stepwise filtering ensures that the analysis focuses only on the target population relevant to occupational injury assessment. Pakistan’s construction injury data for all LFS survey years from 2001–02 to 2017–18 are provided in the Appendix for reference.

### Explanation of variables

The study defines the variables as follows:

1. Age: The age of injured construction workers is categorized into six groups: 10–20, 21–30, 31–40, 41–50, 51–60, and over 60 years. This classification enables analysis of how injury risk varies across different stages of working life, highlighting whether younger or older workers are more vulnerable to occupational hazards in the construction sector.
2. Province: This variable is categorized into all four provinces: Punjab, Sindh, Khyber Pakhtunkhwa (KP), and Balochistan, because these regions collectively encompass the majority of Pakistan’s population and construction workforce. Including all four ensures representative coverage of the country’s construction sector and allows for meaningful comparisons of occupational injury patterns across different geographic and socioeconomic contexts.
3. Area: Injured construction workers are classified based on their place of working—urban or rural. This distinction allows analysis of how working in different environments may influence exposure to occupational hazards, access to healthcare, and overall injury risk.
4. Education: The educational background of injured construction workers is categorized into four groups: No education (no formal schooling), Primary (up to 5 years of schooling), Secondary (up to 10 years), and Intermediate and above (more than 10 years of education). Workers with higher qualifications, such as graduates or postgraduates, are included in the Intermediate and above category because they constitute less than one percent of the injured workforce. This classification helps to assess whether educational attainment influences vulnerability to occupational injuries, as workers with lower education levels may have limited awareness of safety practices and higher exposure to hazards.
5. Occupation: The study categorizes workers according to the Pakistan Standard Classification of Occupations [21], which defines nine occupational groups: Managers, professionals, technicians, clerical support workers, service and sales workers, skilled agricultural, forestry, and fishery workers, Craft and related trade workers, plant/machine operators and assemblers, and elementary occupations. For analytical purposes, these are further grouped into White-collar workers (the first five groups) and Blue-collar workers (the last four groups). The blue-collar group is subdivided into high-skilled (skilled agricultural, forestry, and fishery workers and craft and related trade workers) and low-skilled (plant/machine operators and assemblers and elementary occupations). This classification, previously employed by Dumont (2006) [22], allows the study to examine how skill level and type of occupation influence vulnerability to occupational injuries, with low-skilled workers often facing higher exposure to hazardous conditions.
6. Unsafe act: In the LFS, construction workers’ unsafe behaviors are recorded under 13 specific options, which are consolidated into eight main categories for analysis. Smaller or less frequent behaviors are grouped under “Other.” The main categories include excess speed, horseplay, failure of safety devices, using unsafe equipment or using equipment unsafely, taking unsafe positions, failure to use provided personal protective equipment (PPE), unsafe loading or stacking, and other. This classification helps identify the most common worker actions that contribute to occupational injuries and highlights areas where targeted safety training and preventive measures are needed.
7. Treatment: The LFS records the type of medical or recovery response received by injured construction workers. There are four options: hospitalized, consulted a doctor, nurse, or other medical professional, took time off work, or none (no treatment). This classification helps to understand the severity of injuries, the burden on healthcare and work productivity, and the recovery needs of workers, which can guide occupational health policies and preventive measures in the construction sector.
8. Unsafe condition: The LFS records the workplace factors that contribute to construction accidents. Ten categories are identified, including unguarded or inadequately guarded equipment, defective tools or materials, unsafe design or construction, poor illumination, inadequate ventilation, improper clothing or footwear, lack of necessary protective equipment, poor housekeeping, slippery surfaces, and other conditions. These categories help to pinpoint hazards in the work environment, identify areas needing intervention, and inform targeted safety measures to prevent occupational injuries.

### Trends in index values

This study examines the trends in index values related to the characteristics of occupationally injured construction workers in Pakistan. The survey was extended in 2001–02, serving as the reference year for the analysis. The PBS began gathering information on occupational injuries in the LFS starting from 2001–02, which provides the baseline dataset for this study. The general linear time trend model is used to explain the relationship between a dependent variable and an explanatory variable, as outlined in Equation (1):


$$Y_{t}=\beta_{1}+\beta_{2}t+\varepsilon_{t}.$$ 1

In this context, *Y* represents the socioeconomic or demographic characteristics of construction workers who have been injured, and t represents the explanatory variable, with each variable’s reference year. $\beta_{1}$ stands for the intercept, $\beta_{2}$ represents the slope, and ε accounts for the error term. This trend analysis provides useful information for policymakers by enabling observation of changes since the reference year. The base year, 2001–02, is standardized to 100 to allow comparison over time. The linear slope (*β*₂) is then estimated for each trend to indicate its direction and magnitude. An upward slope reflects a worsening condition, while a downward slope indicates improvement. Red arrows (↑) represent upward trends, whereas green arrows (↓) denote downward trends.

## Results

Table 1 presents the index values of occupational injuries among construction workers in Pakistan, categorized by age groups across four provinces from 2001–02 to 2017–18, with 2001–02 as the reference year (index = 100). The table highlights how injury patterns vary by age over time. Younger workers aged 10–20 and 21–30 years show an overall increasing trend in injuries, indicated by positive *β* values (2.1 and 1.5, respectively). This suggests that these age groups are increasingly exposed to occupational hazards, possibly due to their higher involvement in physically demanding and risky tasks, limited work experience, or lower awareness of safety measures. The peak index values for these groups occur between 2007–08 and 2010–11, reflecting periods of inadequate enforcement of safety practices. Workers aged 31–40 and 41–50 years show a slight declining trend (*β* = −1.1 and −1.0, respectively), indicating a modest reduction in injuries over the study period. This may be due to greater experience, better familiarity with safe practices, or assignment to less hazardous tasks. The 51–60 and more than 60 years age groups display a more pronounced downward trend (*β* = −3.2 and −2.5), with index values fluctuating significantly but generally lower than the reference year. This could be explained by reduced physical involvement in hazardous activities, transition to supervisory roles, or underreporting of injuries among older workers. Overall, the data reveal that younger, less experienced construction workers face higher risks of occupational injuries, while older workers have lower but still significant exposure. These patterns underscore the need for age-targeted safety interventions and training programs in the construction sector.

**Table 1:** Index values of occupational injuries by age among construction workers across four provinces of Pakistan (2001–02 to 2017–18).

AGE	2001–02	2003–04	2005–06	2006–07	2007–08	2008–09	2009–10	2010–11	2012–13	2013–14	2014–15	2017–18	*β*	TREND
10–20 years	100.0	68.4	90.2	116.6	135.7	102.2	115.0	133.8	130.0	97.2	108.9	109.7	2.1	↑
21–30 years	100.0	57.1	83.1	79.4	99.6	86.4	106.4	88.7	89.2	104.2	104.1	82.7	1.5	↑
31–40 years	100.0	131.5	124.1	111.2	95.1	111.8	80.9	101.1	97.1	104.6	104.2	113.0	−1.1	↓
41–50 years	100.0	133.2	118.0	120.3	76.3	95.4	108.7	100.7	116.1	103.0	87.9	114.2	−1.0	↓
51–60 years	100.0	155.6	94.4	69.7	105.8	109.8	99.5	84.1	73.8	72.8	80.1	96.2	−3.2	↓
>60 years	100.0	127.6	43.0	143.5	33.6	138.3	54.2	65.0	72.9	107.5	98.6	50.0	−2.5	↓

*Source*: Authors’ calculation from LFS, 2001–02 to 2017–18.

Table 2 shows the index values of occupational injuries by province over the period 2001–02 to 2017–18. The data highlight clear differences in injury trends across the four provinces. Punjab exhibits a slight downward trend (*β* = −0.6), with injuries fluctuating over the years but returning near the reference level by 2017–18. This reflect more stable construction practices, better access to safety measures, or stronger regulatory enforcement compared to other provinces. Sindh shows no significant overall trend, with injuries varying year to year, possibly due to inconsistent safety compliance and rapid urban construction growth. KP displays a pronounced upward trend (*β* = 4.6), with the index rising to 183.9 by 2017–18, suggesting increasing construction activity, more hazardous work conditions, or limited safety oversight in the province. In contrast, Balochistan shows a sharp decline (*β* = −4.4), with the index falling to 39.1, which may reflect lower construction activity, smaller workforce, or underreporting of injuries in this sparsely populated province. Overall, the table indicates that provincial differences in construction activity levels, workforce size, regulatory enforcement, and reporting practices contribute to variation in occupational injury patterns.

**Table 2:** Index values of occupational injuries by province and area among construction workers across four provinces of Pakistan (2001–02 to 2017–18).

PROVINCE	2001–02	2003–04	2005–06	2006–07	2007–08	2008–09	2009–10	2010–11	2012–13	2013–14	2014–15	2017–18	*β*	TREND
Punjab	100.0	110.7	87.1	100.9	96.7	94.4	89.9	91.9	100.5	94.2	86.5	100.9	−0.6	↓
Sindh	100.0	83.4	105.5	71.5	111.2	103.6	117.6	107.7	85.9	106.0	108.6	71.7	0.0	–
KP	100.0	85.9	178.2	166.0	108.3	140.2	129.8	138.8	151.7	130.7	164.5	183.9	4.6	↑
Balochistan	100.0	96.1	13.2	108.9	25.6	24.9	20.6	42.7	29.9	42.3	47.3	39.1	−4.4	↓

*Source*: Authors’ calculation from LFS, 2001–02 to 2017–18.

Table 3 shows the index values of occupational injuries among construction workers in Pakistan, categorized by area. Urban workers exhibit a declining trend in injuries over time (*β* = −2.1), despite several peaks between 2003–04 and 2007–08. The higher index values in the early years suggest that urban construction sites may have initially posed greater hazards due to factors such as higher project density, complex infrastructure tasks, use of advanced machinery, and greater exposure to industrial activities. The subsequent decline could indicate gradual improvements in urban safety practices, increased enforcement of OSH regulations, or better access to safety equipment and training. Rural workers, on the other hand, show a slight upward trend (*β* = 0.6), with the index value reaching 100.4 by 2017–18. The initially lower index values in rural areas suggest that construction activities were less intensive or hazardous compared to urban areas. However, the gradual increase over time may reflect rising construction activity in rural regions, expansion of infrastructure projects, or limited availability of safety measures, training, and supervision in these areas. The table indicates that urban construction workers faced higher injury rates historically, but improvements over time have reduced risks, while rural workers have seen a slow increase in injuries, highlighting the need for targeted safety interventions across both areas.

**Table 3:** Index values of occupational injuries by area (urban vs. rural) among construction workers across four provinces of Pakistan (2001–02 to 2017–18).

AREA	2001–02	2003–04	2005–06	2006–07	2007–08	2008–09	2009–10	2010–11	2012–13	2013–14	2014–15	2017–18	*β*	TREND
Urban	100.0	135.6	138.8	141.0	161.6	120.7	128.0	122.5	104.0	130.2	109.1	98.8	−2.1	↓
Rural	100.0	89.3	88.3	87.7	81.4	93.8	91.6	93.2	98.8	90.9	97.3	100.4	0.6	↑

*Source*: Authors’ calculation from LFS, 2001–02 to 2017–18.

Table 4 shows the index values of occupational injuries among construction workers by educational attainment. Workers with no formal education consistently show a declining trend (*β* = −1.8), which may reflect lower participation in complex construction tasks or potential underreporting of injuries. Workers with primary and secondary education display moderate upward trends (*β* = 4.2 and 0.8), indicating that as workers engage in slightly more skilled activities, their exposure to hazards increases. In contrast, workers with intermediate education or higher experience a sharp and sustained rise in injury indices (*β* = 39.2), likely due to their involvement in technical, supervisory, or specialized roles where both physical and psychosocial risks are greater, and reporting may be more complete. These patterns suggest that higher-educated construction workers, despite greater knowledge or awareness, face higher occupational risks, underscoring the need for targeted OSH interventions and training tailored to the educational profile of the workforce.

**Table 4:** Index values of occupational injuries by educational attainment among construction workers across four provinces of Pakistan (2001–02 to 2017–18).

EDUCATION	2001–02	2003–04	2005–06	2006–07	2007–08	2008–09	2009–10	2010–11	2012–13	2013–14	2014–15	2017–18	*β*	TREND
No education	100.0	90.6	81.0	90.7	82.5	71.9	81.5	86.4	83.6	84.8	72.6	67.3	−1.8	↓
Primary	100.0	142.5	144.2	121.6	134.0	163.8	149.8	128.9	148.9	150.1	176.7	168.2	4.2	↑
Secondary	100.0	87.7	112.6	101.8	117.4	116.1	93.8	109.6	99.7	91.6	102.7	126.9	0.8	↑
Intermediate and above	100.0	139.5	367.4	434.9	386.0	679.1	876.7	395.3	355.8	481.4	602.3	683.7	39.2	↑

*Source*: Authors’ calculation from LFS, 2001–02 to 2017–18.

Table 5 presents the index values of occupational injuries among construction workers, categorized by occupation. White-collar workers show extremely high fluctuations in the early years, peaking at over 2700 in 2005–06, but decline dramatically to near zero in 2014–15, resulting in a strong negative trend (*β* = −99.4). This pattern likely reflects very small numbers of reported injuries among white-collar staff, making index values highly sensitive to minor changes, rather than actual risk. Blue-collar workers, who constitute the majority of the construction workforce, show relatively stable index values around 100, indicating consistent exposure to occupational hazards over time, with a very slight overall decline (*β* = −0.1). Within the blue-collar category, high-skilled workers (e.g., skilled trades, craft workers) experience a modest decreasing trend (*β* = −0.4), while low-skilled workers (e.g., machine operators, elementary occupations) show a slight upward trend (*β* = 0.5), suggesting that low-skilled workers are increasingly exposed to injury risks. These results highlight that the bulk of construction injuries occur among blue-collar workers, particularly those performing low-skilled tasks, emphasizing the need for targeted OSH interventions for this group.

**Table 5:** Index values of occupational injuries by occupation among construction workers across four provinces of Pakistan (2001–02 to 2017–18).

OCCUPATION	2001–02	2003–04	2005–06	2006–07	2007–08	2008–09	2009–10	2010–11	2012–13	2013–14	2014–15	2017–18	*β*	TREND
White-collar	100.0	2030.8	2753.8	1053.8	353.8	1330.8	1792.3	315.4	930.8	253.8	0.0	792.3	−99.4	↓
Blue-collar	100.0	97.5	96.5	98.7	99.7	98.4	97.8	99.7	98.9	99.8	100.1	99.1	−0.1	↓
High-skilled	100.0	102.1	85.7	107.5	106.3	117.4	96.9	77.9	90.4	98.9	98.4	100.4	−0.4	↓
Low-skilled	100.0	94.7	103.0	93.5	95.7	87.1	98.4	112.7	104.0	100.3	101.1	98.3	0.5	↑

*Source*: Authors’ calculation from LFS, 2001–02 to 2017–18.

Table 6 presents the index values by unsafe acts from 2001–02 to 2017–18. The data reveal that certain unsafe behaviors consistently contribute to injuries and show distinct trends over time. Horseplay exhibits a substantial increase, with the index rising from 100 to 732.7 and a strong positive trend (*β* = 49.7), indicating that playful or reckless behavior on sites increasingly leads to injuries. Similarly, failure to use protective equipment shows a pronounced upward trend (*β* = 14.0), reflecting ongoing noncompliance with safety protocols, despite interventions. Unsafe equipment (*β* = 3.7), failure of safety devices (*β* = 7.9), and unsafe positions (*β* = 1.8) also show modest increases, suggesting persistent risks associated with improper handling, faulty machinery, or hazardous working postures. In contrast, excess speed (*β* = 0.0) remains relatively stable, indicating limited contribution to overall injuries, while unsafe loading/stacking (*β* = −3.8) and other minor unsafe acts (*β* = −7.2) show declining trends, possibly due to improved training or enforcement in these areas. The table highlights that behavioral factors, particularly horseplay and nonuse of protective equipment, are major contributors to occupational injuries in construction, emphasizing the need for targeted behavioral interventions and stricter enforcement of safety compliance on worksites.

**Table 6:** Index values of occupational injuries by unsafe acts among construction workers across four provinces of Pakistan (2001–02 to 2017–18).

UNSAFE ACT	2001–02	2003–04	2005–06	2006–07	2007–08	2008–09	2009–10	2010–11	2012–13	2013–14	2014–15	2017–18	*β*	TREND
Excess speed	100.0	27.9	95.9	117.4	95.4	79.8	64.6	69.7	67.9	88.1	78.0	95.4	0.0	–
Horseplay	100.0	347.7	405.6	301.9	426.2	629.9	498.1	794.4	596.3	746.7	615.9	732.7	49.7	↑
Failure of safety devices	100.0	14.2	137.2	146.6	222.4	185.2	211.0	213.1	133.0	165.8	161.0	173.4	7.9	↑
Unsafe equipment	100.0	191.7	137.6	165.1	118.1	173.7	238.7	197.2	188.1	188.4	166.5	147.7	3.7	↑
Unsafe position	100.0	97.5	89.1	82.5	105.8	99.6	107.9	106.0	118.5	104.8	116.1	103.9	1.8	↑
Fail to use protective equipment	100.0	554.2	188.1	427.1	101.7	345.8	288.1	252.5	833.9	578.0	50.8	406.8	14.0	↑
Unsafe loading/stacking	100.0	203.2	145.9	105.7	112.5	64.5	89.1	154.1	107.7	63.0	115.5	110.5	−3.8	↓
Others	100.0	119.1	71.7	57.5	39.7	47.6	25.1	13.5	30.0	27.5	38.3	29.3	−7.2	↓

*Source*: Authors’ calculation from LFS, 2001–02 to 2017–18.

Table 7 shows the index values based on the type of treatment received. The data reveal trends in how injured workers were treated over time and provide insights into injury severity and workplace response. Hospitalization shows a declining trend (*β* = −4.5), decreasing from 100 to 89.6, indicating that fewer workers required inpatient care over time, which may reflect either a shift toward less severe injuries or increased outpatient care options. Consultation with a doctor, nurse, or other medical professional also slightly declines (*β* = −0.4), suggesting that medical attention for minor injuries remained relatively stable but did not increase proportionally with injury numbers. Time off work shows a positive trend (*β* = 2.5), rising from 100 to 128.6, reflecting that many workers needed extended recovery periods following injuries, likely due to physical severity or workplace policies requiring rest. The “None” category, representing workers who did not receive any treatment, also shows a small increase (*β* = 2.2), which may indicate gaps in immediate medical access or underreporting of minor injuries. The table emphasizes that while severe cases requiring hospitalization are declining, injuries still impose significant time costs on workers, highlighting the importance of preventive measures, timely medical care, and workplace safety interventions.

**Table 7:** Index values of occupational injuries by treatment among construction workers across four provinces of Pakistan (2001–02 to 2017–18).

TREATMENT	2001–02	2003–04	2005–06	2006–07	2007–08	2008–09	2009–10	2010–11	2012–13	2013–14	2014–15	2017–18	*β*	TREND
Hospitalized	100.0	102.2	94.5	105.9	76.9	84.9	74.6	62.9	77.1	41.0	34.4	89.6	−4.5	↓
Consultation	100.0	128.8	134.8	114.2	94.9	109.2	108.1	107.3	96.5	114.8	126.7	108.1	−0.4	↓
Time off work	100.0	108.9	102.7	130.2	158.6	153.7	138.3	143.5	134.3	152.2	118.3	128.6	2.5	↑
None	100.0	22.8	19.4	37.2	79.7	44.5	66.9	72.8	95.9	63.3	67.7	64.4	2.2	↑

*Source*: Authors’ calculation from LFS, 2001–02 to 2017–18.

Table 8 presents the index values based on the type of unsafe conditions present at the workplace. The table provides insights into which unsafe conditions were most associated with injuries and how these trends evolved over time. Unguarded or inadequately guarded equipment shows a declining trend (*β* = −8.2), decreasing from a peak of 309.9 in 2005–06 to 106.3 in 2017–18, suggesting improvements in machine guarding or safety awareness over time. Defective tools and unsafe design show upward trends (*β* = 5.3 and 5.8, respectively), indicating persistent risks from poorly maintained equipment and construction design flaws. Poor illumination also increased (*β* = 5.5), highlighting continued hazards related to inadequate lighting on worksites. Inadequate ventilation and improper clothing/footwear show declines (*β* = −3.0 and −4.1), reflecting partial mitigation of these hazards, while lack of protective equipment slightly increased (*β* = 1.5), suggesting gaps in PPE provision or compliance. Slippery surfaces also show a rising trend (*β* = 5.4), indicating a recurring hazard that contributes to slips, trips, and falls. Poor housekeeping and other miscellaneous unsafe conditions declined (*β* = −4.4 and −7.3), pointing to improved general site organization and reduction of uncommon hazards. These patterns indicate that while some unsafe conditions have been addressed over time, others, such as defective tools, unsafe designs, poor lighting, and slippery surfaces, remain significant contributors to occupational injuries, emphasizing the need for continued preventive measures and strict enforcement of safety protocols.

**Table 8:** Index values of occupational injuries by unsafe condition among construction workers across four provinces of Pakistan (2001–02 to 2017–18).

UNSAFE CONDITION	2001–02	2003–04	2005–06	2006–07	2007–08	2008–09	2009–10	2010–11	2012–13	2013–14	2014–15	2017–18	*β*	TREND
Unguarded/inadequately guarded	100.0	242.4	309.9	245.7	203.2	250.1	130.1	140.0	154.7	160.0	174.1	106.3	−8.2	↓
Defective tool	100.0	130.6	115.5	148.6	139.2	210.9	180.8	149.6	138.8	176.0	172.6	170.3	5.3	↑
Unsafe design	100.0	101.0	121.0	125.8	169.4	118.8	153.5	210.6	173.0	163.8	178.8	124.1	5.8	↑
Poor illumination	100.0	85.0	120.4	79.2	85.8	75.4	159.2	98.3	102.1	110.4	128.3	193.8	5.5	↑
Inadequate ventilation	100.0	40.4	41.4	6.1	21.2	52.0	23.7	35.9	18.2	0.0	47.0	36.9	−3.0	↓
Improper clothing and footwear	100.0	141.7	163.5	100.4	166.6	118.5	151.9	163.5	152.1	98.8	32.6	97.5	−4.1	↓
No protection equipment	100.0	63.5	62.1	86.1	85.7	64.4	86.8	85.2	104.0	69.4	65.3	123.7	1.5	↑
Poor housekeeping	100.0	27.4	83.0	127.8	87.2	61.2	20.1	38.7	35.5	56.3	88.0	12.2	−4.4	↓
Slippery surfaces	100.0	68.8	90.5	90.4	104.8	90.4	133.7	116.7	125.2	139.5	118.9	146.2	5.4	↑
Other	100.0	110.7	55.6	41.4	20.1	29.5	12.6	9.9	22.3	14.8	21.0	21.5	−7.3	↓

*Source*: Authors’ calculation from LFS, 2001–02 to 2017–18.

## Discussion

The results of this study provide new insights into OSH vulnerabilities among construction workers in Pakistan, with significant implications for both worker welfare and infrastructure development. Analysis of LFS data indicates that younger workers, rural laborers, and low-skilled, less-educated blue-collar employees face disproportionately higher risks of workplace injuries. These groups constitute a substantial portion of the labor force engaged in large-scale infrastructure projects. Injuries are more prevalent among younger workers, who tend to take greater risks and are less likely to adhere to safety measures compared to their older counterparts, reflecting differences in safety attitudes and experience. Punjab reports the highest incidence of construction injuries despite provincial safety legislation. Variations across provinces reflect differences in workforce size, urban–rural distribution, construction activity, and reporting practices: Punjab shows a stable or slightly declining trend, Sindh exhibits mixed trends due to rapid urban growth, KP has a rising trend linked to increased construction and reporting, and Balochistan shows lower, declining numbers. These patterns underscore the need for provincial OSH policymaking, including enforcing the Pakistan Labour Policy (2010),^2^ strengthening regulations, and implementing targeted interventions such as safety training, inspections, and standardized injury reporting to promote safer construction practices.

Workplace location further contributes to vulnerability. Rural workers face elevated risks due to limited access to healthcare facilities, safety training, and lower educational attainment. Workers with minimal education have reduced safety awareness, emphasizing the need for safety culture initiatives and public awareness campaigns through mass media. The observed decline in injury index values among construction workers with no education does not necessarily indicate improved safety conditions; instead, it likely reflects structural and reporting factors. Over time, less-educated workers may have shifted toward informal or relatively lower-risk tasks, or may be underrepresented in survey data due to underreporting. In contrast, more educated workers (primary and above) are more often engaged in skilled or technical construction roles that involve greater exposure to machinery and hazardous activities, leading to higher recorded injury incidence. Moreover, better awareness and reporting practices among educated workers may also contribute to the higher observed injury rates. Unsafe acts remain key contributors to injuries. Unsafe conditions can be categorized as failure to recognize hazards, deliberate engagement in unsafe activities, and unsafe actions within hazardous situations. The observed positive trend in the percentage of time off work reflects both longer recovery periods and higher injury severity among construction workers. Despite some improvements, construction injuries continue to impose significant socioeconomic costs, including medical expenses, wage losses, and reduced quality of life. Many injuries remain unreported. The lack of an effective labor inspection system exacerbates these vulnerabilities, as noted by the ILO. Most injured workers often employed long hours, engaged in subsidiary work, and lacked formal contracts or regular pay. The ILO continues to work with Pakistan and other countries to strengthen OSH frameworks through conventions and targeted measures [11].

Embedding OSH measures into infrastructure planning and policymaking is crucial. Traditional safety regulations are insufficient to address systemic vulnerabilities, particularly in rural and informal labor markets. Expanding social protection, strengthening provincial labor laws, and mandating OSH compliance in different sector contracts can enhance workforce resilience. These interventions improve project efficiency and support sustainable infrastructure development. While this study focuses on Pakistan, the findings are applicable to other developing economies undergoing rapid economic transitions. Vulnerable construction labor is a common challenge across developing countries. Pakistan’s experience underscores the need for international organizations, governments, and industry stakeholders to integrate OSH as a central component of infrastructure management, safeguarding both human welfare and long-term project sustainability.

## Conclusion

### Key findings

This study examined OSH vulnerabilities among construction workers in Pakistan using 12 waves of LFS data from 2001–02 to 2017–18. The analysis revealed that workplace injuries were disproportionately concentrated among younger and rural workers, and low-skilled blue-collar employees with limited education. Unsafe acts, including neglecting the use of protective equipment, and hazardous site conditions, such as defective tools, unsafe designs, poor scaffolding, and slippery surfaces, remained primary contributors to injuries, highlighting persistent structural weaknesses in workplace safety practices within the construction sector.

The findings indicate that younger workers are more prone to injuries due to their limited experience, lower safety awareness, and higher propensity for risk-taking behavior. Rural workers are particularly vulnerable because of restricted access to occupational safety training, healthcare facilities, and adequate supervision, while low-skilled and less-educated laborers are more likely to engage in high-risk tasks without proper guidance, reflecting the close association between occupational risk and worker capability. Unsafe behaviors and hazardous workplace conditions act synergistically to increase accident prevalence, reinforcing the need for both behavioral interventions and structural improvements in safety management.

### Implications for construction and infrastructure development

Construction labor constitutes the backbone of infrastructure development in Pakistan, particularly in development projects. High rates of occupational injuries can therefore have significant operational and economic repercussions, including reduced labor productivity, increased medical and insurance costs, and interruptions to project schedules. Such disruptions may translate into cost overruns and lower quality of infrastructure delivery, underscoring that worker safety is not only a matter of labor welfare but also a critical element of effective project management and long-term infrastructure sustainability.

### Policy recommendations

Policy measures derived from this study emphasize multilevel interventions. Targeted safety training programs are essential for young, rural, and low-educated workers to improve awareness of workplace hazards and promote safer practices. Strict enforcement of PPE and adherence to site safety protocols must be maintained through robust monitoring and accountability mechanisms. Regular workplace inspections and safety audits are vital to identify and rectify hazardous conditions before incidents occur. Expanding access to occupational health services, medical support, and compensation schemes can reduce the financial burden on injured workers and facilitate quicker recovery and return to work. Furthermore, formalization of employment contracts and integration of OSH compliance into national labor laws and infrastructure procurement policies can improve accountability, working conditions, and the overall safety culture within the sector.

### Limitations

Despite its contributions, this study has several limitations. First, the reliance on secondary data from the LFS may result in underreporting of occupational injuries, particularly within informal employment settings where a substantial portion of construction work occurs. Informal and subcontracted labor often operate outside formal monitoring systems, and their injuries may not be fully captured, potentially underestimating the true burden of workplace hazards. Additionally, the LFS does not list the construction industry separately; blue-collar occupations are categorized only as high-skilled (skilled agricultural, forestry, and fishery workers, and craft and related trade workers) or low-skilled (plant/machine operators, assemblers, and elementary occupations), limiting the ability to analyze construction-specific risks.

Second, the available dataset does not provide detailed information on the timing of accidents, such as the observation that most incidents occurred in the morning [23], which limits the ability to analyze patterns associated with work schedules, fatigue, or shift-specific risks, factors shown in the literature to influence occupational injuries. Additionally, the dataset lacks information on workplace-specific conditions, employer-level safety management practices, and the frequency of medical or fitness examinations. Consequently, it is not possible to determine whether workers are regularly assessed for their capacity to perform physically demanding tasks, often at heights or under difficult climatic conditions, as recommended in prior studies. These limitations constrain the study’s ability to evaluate the effectiveness of preventive mechanisms, the role of safety training, and the influence of organizational safety culture on injury outcomes.

Third, the absence of information on fatalities and affected body parts in LFS waves after 2006–07 limits the evaluation of injury severity and long-term health consequences. Earlier survey waves provided these critical indicators, enabling the assessment of the most vulnerable worker groups, the type of injuries sustained, and potential permanent disabilities. Fatality data, in particular, are crucial for benchmarking Pakistan’s OSH performance against international standards and for informing evidence-based policy and regulatory interventions [2, 15].

Finally, the cross-sectional nature of the LFS data precludes tracking individual workers over time. As a result, the study cannot capture cumulative exposure, repeated injuries, or long-term occupational health outcomes.

### Future research directions

Future research should incorporate updated datasets and complement secondary data with primary surveys or field-based studies to capture information on workplace environments, safety culture, and behavioral factors influencing accidents. Studies could also explore regional disparities in safety enforcement, assess the impact of technological innovations such as automated safety monitoring and wearable protective devices, and evaluate the effectiveness of training and regulatory interventions. Expanding the scope to include longitudinal tracking of occupational injuries and their socioeconomic impacts would provide more comprehensive evidence for policy. Such research is critical for developing targeted, evidence-based strategies to strengthen OSH, improve labor productivity, and enhance the resilience and sustainability of Pakistan’s construction and infrastructure sectors.

### Global relevance

Beyond the national context, the findings hold relevance for other developing economies undergoing rapid urbanization and energy transitions. Countries in South Asia, Africa, and Latin America face similar challenges in managing a vulnerable construction workforce while meeting ambitious infrastructure goals. Lessons from Pakistan emphasize the need for governments, industry stakeholders, and international organizations to treat OSH as an integral component of infrastructure planning and project delivery. Protecting the workforce not only safeguards human well-being but also ensures the reliability, efficiency, and long-term sustainability of critical infrastructure projects globally.

## Data Availability

The datasets analyzed during the current study are available from the Pakistan Bureau of Statistics. https://www.pbs.gov.pk/.

## Notes

Click the Tableau Public link to see the interactive visualization. Hovering over the map will show the comparison of sectoral fatalities. Please note that this is within sectoral injuries of selected sectors, and the sum is not equal to 100%. https://public.tableau.com/app/profile/injuries.in.construction/viz/ConstructionFatalities_16284312021940/Dashboard1?publish=yes.

Labour Policy 2010, Government of Pakistan. https://www.numl.edu.pk/downloads/16673773818.2.2%20-4-6-7%29%20Government%20of%20Pakistan%20Labour%20Policy%202010.pdf.

## Additional Files

The additional files for this article can be found as follows:

Supplementary Appendix. Construction Injuries Among Workers in Pakistan Across LFS Survey Waves (2001–02 to 2017–18).
