# Supplementary material for: Workforce Safety Vulnerabilities in Pakistan’s Construction Sector
Source: Ann Glob Health. 2026 Apr 15;92(1):32. doi: 10.5334/aogh.5077 (PMC13089374; doi:10.5334/aogh.5077)
Supplement: Supplementary Appendix. — Construction Injuries Among Workers in Pakistan Across LFS Survey Waves (2001–02 to 2017–18). [file agh-92-1-5077-s1.pdf]

Appendix. Construction Injuries Among Workers in Pakistan Across LFS Survey Waves (2001-02 to 2017-18)

LFS Survey Year	Number of Construction Injuries
2001-02	151
2003-04	109
2005-06	243
2006-07	191
2007-08	201
2008-09	284
2009-10	309
2010-11	329
2012-13	381
2013-14	434
2014-15	497
2017-18	516

Note: Values were extracted from different waves of the LFS

#
